# Equivalence between positive and negative refractive index materials in electrostatic cloaks

**DOI:** 10.1038/s41598-021-00124-w

**Published:** 2021-10-14

**Authors:** Xingcai Li, Juan Wang, Jinghong Zhang

**Affiliations:** 1 School of Physics and Electronic-Electrical Engineering, Ningxia University, Yinchuan, 750021 China; 2 Ningxia Key Laboratory of Intelligent Sensing& Desert Information, Ningxia University, Yinchuan, 750021 China; 3 Xinhua College of Ningxia University, Yinchuan, 750021 China; 4 Department of Civil Engineering, Yancheng Institute of Technology, Yancheng, China

**Keywords:** Applied physics, Electronic properties and materials, Theory and computation

## Abstract

We investigate, both theoretically and numerically, the equivalence relationship between the positive and negative refraction index dielectric materials in electrostatic invisibility cloak. We have derived an analytical formula that enables fast calculate the corresponding positive dielectric constant from the negative refraction index material. The numerical results show that the negative refraction index material can be replaced by the positive refractive index materials in the static field cloak. This offers some new viewpoints for designing new sensing systems and devices in physics, colloid science, and engineering applications.

## Introduction

In military and some scientific experiments, hiding one object from the environmental is a fundamental requirement^1^. Several studies have confirmed that using metamaterials or designing special structures can make the target cloaking or hiding in the environment^2,3^. The transformation optics and scattering cancellation-based cloaking should be two powerful tools^4–6^. The basic idea of transformation optics is to manipulate electromagnetic waves by precisely designing the refractive index and permeability of every point and every direction in space. Scattering cancellation-based cloaking mainly using metamaterial, meta-surfaces, graphene and/or plasmonic materials to eliminate the scattering field of target object, and it can also be used in some physical fields^7–18^. As some of the literatures suggests that each cloaking technique has its own advantages and disadvantages^7,19^, exploring the new methods, for example, the illusion optics, or the common materials that can also make targets invisible from different ways, will significantly promote the practical application of relevant research results^20–22^.

At presents some researchers have reported that the invisibility cloak can be widely used in electromagnetic waves^23–26^, mechanical waves^27^, elastic waves^28,29^, matter waves^30^, water waves^31^, magnetic fields^32^, DC magnetic or electric fields^33–35^, current^36^, and thermal fields^37–39^. Based on the metamaterial, people can also design an electrostatic field concentrator^3^, magnetic field concentrator^40^, asymmetric universal and invisible gateway^41^, the perfect lens^42,43^, perfect transmission channel^44^, general illusion device^21^, transparency coating^45^, dc electric concentrator^46^, tunable invisibility cloaking^47^, special imaging probe^48^ and so on^48–51^. However, the materials used in these devices are anisotropic, negative refractive index medium, chiral materials, even double-negative materials or field gain materials^50,52,53^. Some papers also discuss the tunable electromagnetically induced transparency metamaterial based on solid-state plasma^49^ and the broadband perfect absorption based on plasmonic nanoparticles^54^. It is well known that the negative refractive index materials or metamaterials are hard to be produced, and usually, its sizes are much larger than the targets^55^. Can we find alternatives to negative index materials?

Besides, the electric field commonly exists in the nature, and it may change the object’s physical characteristic^56–58^. Some conditions we need effectively manipulate the electric field, for example, the measurement of electrostatic phenomena in sandstorms and other aerosol weather^59,60^, neuro-medicine ^61^, the shielding of an static electromagnetic field for some special devices or sensors^62^, the enhancement of localized electric field^63^, polymer self-assembly properties induced by strong electric field^64^, etc. In these applications, the placed sensor will undoubtedly cause electric field perturbations, which may affect the experimental results. In contrast, the precise control of the electric field in the target test area helps improve the experimental accuracy. Therefore, it is an interesting and meaningful research topic to design a device that does not notably perturb the applied electric field, but it still keeps the original information of the incident field, or it can adjust the localized field exactly according to our will. Generally speaking, existing designs are based either on anisotropic negative refraction index materials or on the geometric dimensions of the cloak structure^65–67^. At present, material doping has been used to fabricate some new materials with specific properties^68,69^. If the isotropic positive refractive index materials can also be used to design the metamaterials, we think the cloaking will become more useful. Some references have introduced the positive refractive index and isotropic material to induce “invisibility” in the Rayleigh limit for two-dimensional objects^16,67,70,71^. Then, is there an equivalent transformation relationship between positive and negative refractive index materials?

**Figure 1:**
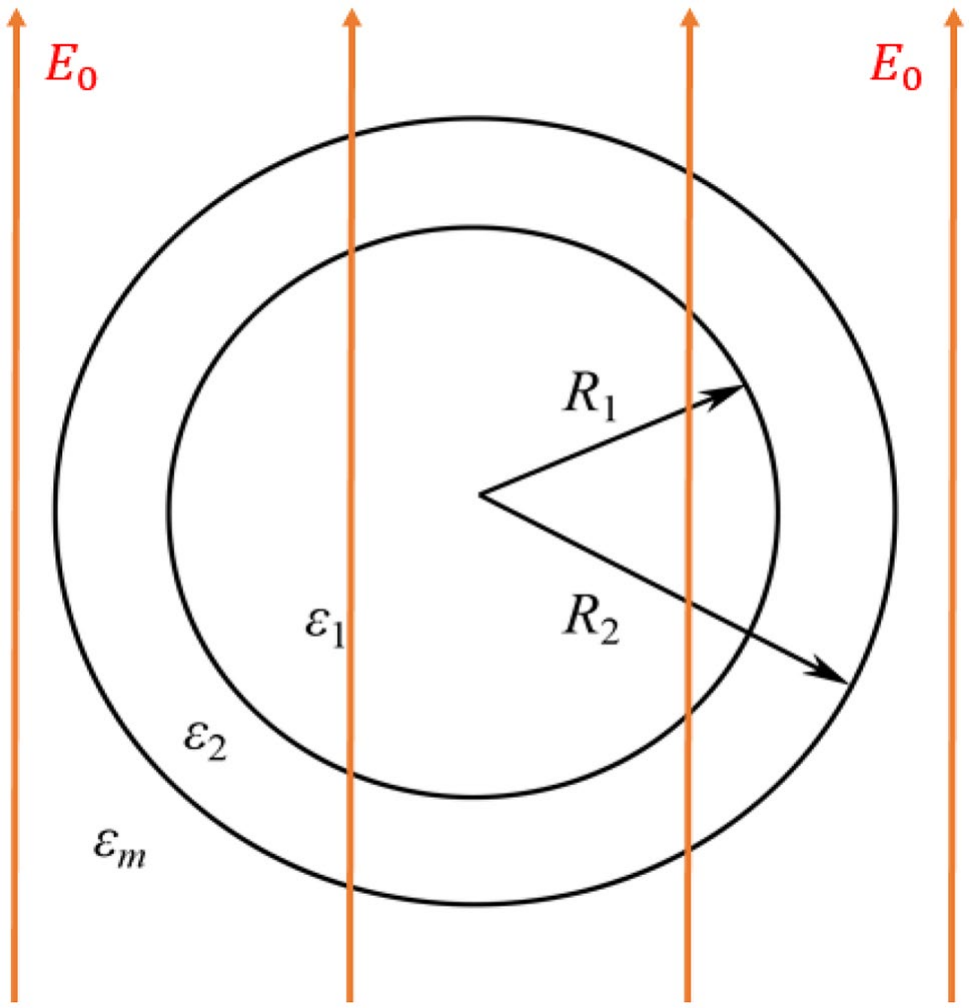
Schematic of a core–shell particle in a uniform electrostatic field.

To answer the above question, we are inspired by the electric potential distribution function of the coated sphere in a uniform static field^58,72–74^. Supposed that the inner and outer radius of a coated sphere are $R_{1}$ and $R_{2}$, respectively, the permittivity in the core and shell are $\varepsilon_{1} ,\varepsilon_{2}$, respectively, and the permittivity of environment medium is $\varepsilon_{m}$, as illustrated in Fig. 1. The applied electric field is along the z-axis, and its intensity is $E_{0}$. For the selected physical model, the core can be thought as the target to be invisible, and the shell zone can be viewed as the functional device that needs to be designed.

The potential inside and outside the core–shell particles can be calculated through the equation $\nabla^{2} \phi { = }0$ with separating variables method, and the equivalent dielectric constant $\varepsilon_{equ}$ of the core–shell particle also can be obtained^73^. We need to make $\varepsilon_{equ} { = }\varepsilon_{m}$, and then a new equation derived,

1 $$\xi (2\varepsilon_{2} + \varepsilon_{m} )\left( {\varepsilon_{1} - \varepsilon_{2} } \right){ = }(\varepsilon_{m} - \varepsilon_{2} )\left( {\varepsilon_{1} + 2\varepsilon_{2} } \right)$$

By solving the above equation and set $\beta = \left( {2\xi + 1} \right)\varepsilon_{1} - \left( {2 + \xi } \right)\varepsilon_{m}$,$\xi = {{R_{1}^{3} } \mathord{\left/ {\vphantom {{R_{1}^{3} } {R_{2}^{3} }}} \right. \kern-\nulldelimiterspace} {R_{2}^{3} }}$, we can get two roots of $\varepsilon_{2}$ for the Eq. (1) we defined them as $\varepsilon_{21}^{a}$ and $\varepsilon_{21}^{b}$, which is expressed as following.

2 $$\varepsilon_{21}^{a} = \frac{{ - \beta + \sqrt {\beta^{2} { + }8\left( {1 - \xi } \right)^{2} \varepsilon_{1} \varepsilon_{m} } }}{{4\left( {1 - \xi } \right)}}$$

3 $$\varepsilon_{21}^{b} = \frac{{ - \beta - \sqrt {\beta^{2} { + }8\left( {1 - \xi } \right)^{2} \varepsilon_{1} \varepsilon_{m} } }}{{4\left( {1 - \xi } \right)}}$$

In addition, we made the two roots $\varepsilon_{21}^{a}$ and $\varepsilon_{21}^{b}$ divided by $\varepsilon_{m}$, then we can obtain the relative permittivity of the shell, and Eqs. (2) and (3) became to a new expression,

4 $$\upvarepsilon _{2}^{{\text{a}}} = \frac{{ - \beta_{1} + \sqrt {\beta_{1}^{2} + 8\left( {1 - \xi } \right)^{2} \varepsilon_{1r} } }}{{4\left( {1 - \xi } \right)}}$$

5 $$\upvarepsilon _{{2}}^{{\text{b}}} = \frac{{ - \beta_{1} - \sqrt {\beta_{1}^{2} { + }8\left( {1 - \xi } \right)^{2} \varepsilon_{1r} } }}{{4\left( {1 - \xi } \right)}}$$

here $\beta_{1} = \left( {2\xi + 1} \right)\varepsilon_{1r} - \left( {2 + \xi } \right)$,$\varepsilon_{1r} { = }{{\varepsilon_{1} } \mathord{\left/ {\vphantom {{\varepsilon_{1} } {\varepsilon_{m} }}} \right. \kern-\nulldelimiterspace} {\varepsilon_{m} }}$ is the relative permittivity of core. The numerical simulation results shown that ${\upvarepsilon }_{{2}}^{{\text{a}}}$ corresponds to an isotropic positive refractive index dielectric material, but $\upvarepsilon _{{2}}^{{\text{b}}}$ corresponds to the negative refractive index material, which means there is a reciprocity relationship between the positive and negative refraction index dielectric materials. Considering the similarity of the two expressions in Eqs. (4) and (5), and add these two equations together. Through some simplify calculation, we can obtain the following formula,

6 $$\upvarepsilon _{{2}}^{{\text{a}}} = \frac{{\left( {2 + \xi } \right) - \left( {2\xi + 1} \right)\varepsilon_{1r} }}{{2\left( {1 - \xi } \right)}} -\upvarepsilon _{{2}}^{{\text{b}}}$$

Therefore, we can calculate the matching positive refractive index according to the negative refractive index for the electrostatic field invisibility cloak. That means if we have obtained the material permittivity of a cloak through the transformation optics or other method, we can deduce its equivalent positive refractive index parameters, which make it much simpler to design a required invisibility structure.

## Results

**Figure 2:**
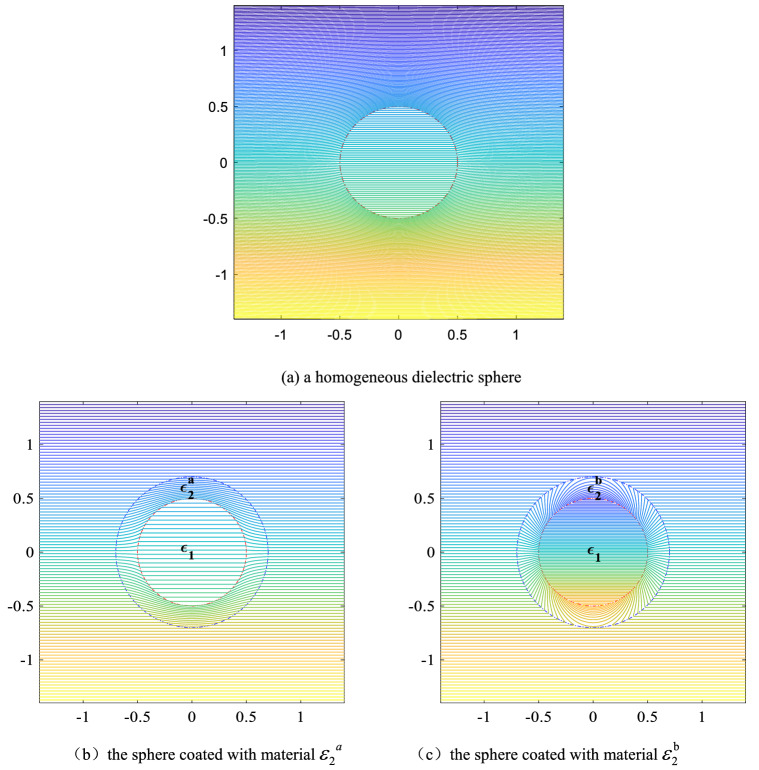
The equipotential line of different spherical object in a uniform electric field.

**Figure 3:**
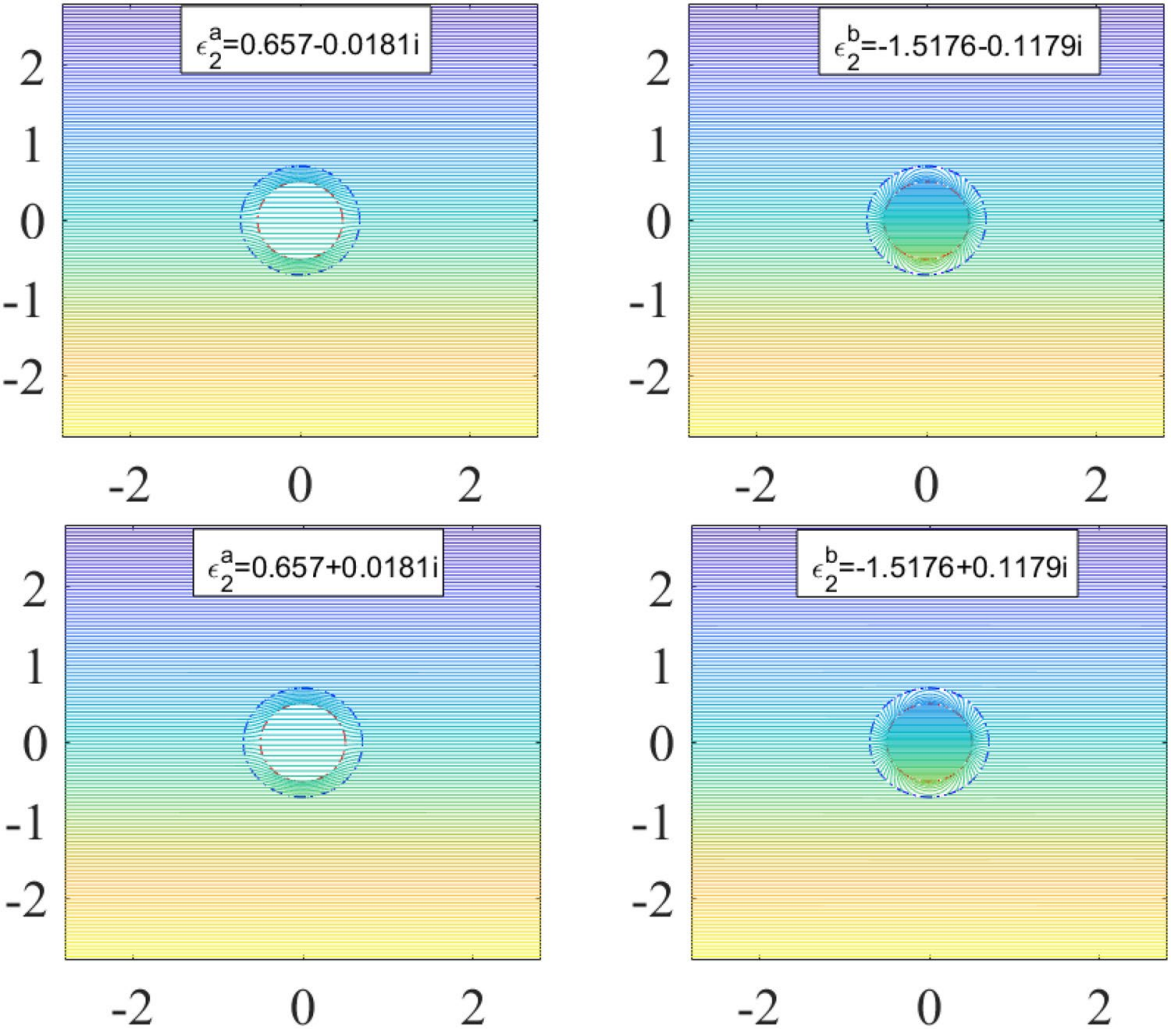
The equipotential line of a layered object with different shell.

To clearly verify the reliability of the above formulas, we calculate the electric potential of the particle surrounded by the designed cloak, whose permittivity is given by the solution of Eq. (4), and the equipotential lines are shown to investigate the perturbation of the particle to the applied electric field. The electric potentials are calculated by Eqs. (7)–(9). Here we set $\varepsilon_{1r} = 2.0 + 0.1i$,$R_{1} = 0.5\;{\text{m}}$, $R_{2} = 0.7\;{\text{m}}$, the thickness of the shell $dr = R_{2} - R_{1}$, $E_{0} = 100\;{\text{V/m}}$. Substitute them into the Eqs. (4) and (5), we can obtain that ${\upvarepsilon }_{{2}}^{{\text{a}}} = 0.6575 - 0.0181i$ and ${\upvarepsilon }_{{2}}^{{\text{b}}} = - 1.5176 - 0.1179i$. The results are shown in Fig. 2. It can be seen from the Fig. 2a that the electric field around the object without the cloak covering are severely distorted, but from Fig. 2b, c we can find there are no any perturbation around the object. It means the spherical object will be perfectly "invisible" in the electric field. Therefore, besides the negative refractive index materials reported by some researchers, the non-negative refractive index materials also can make the object invisible in the electric field. Compared with Fig. 2a, the number of equipotential lines inside the sphere decreases in Fig. 2b (the color becomes lighter), but increases in Fig. 2c (the color becomes darker). So, we conclude that the cloak with permittivity ${\upvarepsilon }_{{2}}^{{\text{a}}}$ can shield the applied electric field to a certain extent, while the cloak with permittivity ${\upvarepsilon }_{{2}}^{{\text{b}}}$ can increase its internal electric potential. Based on these properties, we can design some special devices following the requirement of the experiment. For example, we can make the electrostatic field sensor coated with the material ${\upvarepsilon }_{{2}}^{{\text{a}}}$ to avoid the distortion of the external electric field caused by the sensor, and we also can design a more sensitive sensor with material ${\upvarepsilon }_{{2}}^{{\text{b}}}$ to measure a weak signal. Furthermore, we also find that when the imaginary part of the cloak’s permittivity is positive, the field distribution is the same as that when the imaginary part of cloak’s permittivity is negative, as shown in Fig. 3. So we can conclude that the imaginary part of the medium’s dielectric constant does not affect the response of the medium to the external electrostatic field.

**Figure 4:**
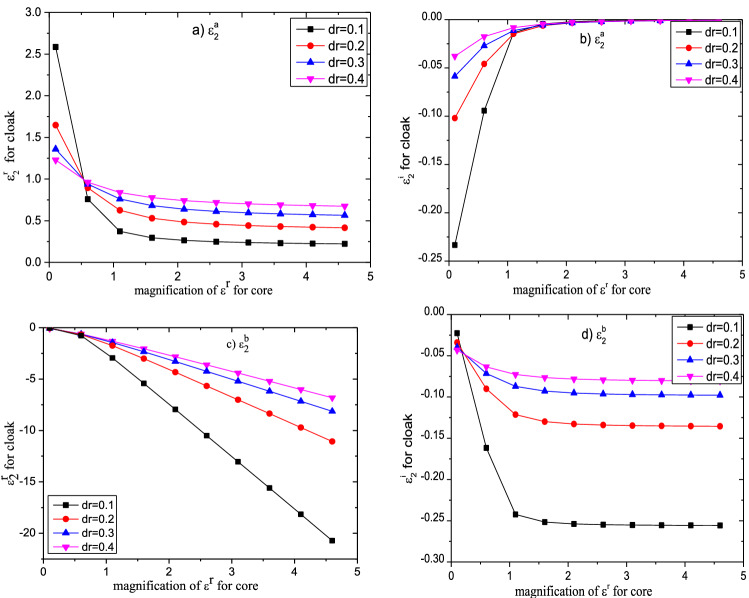
Influence of $\varepsilon^{r}$ on the permittivity of the cloak with different permittivity.

In order to analyze the effect of the core zone on the permittivity of the cloak, we set the permittivity of the core particle can be expressed by $\varepsilon_{1r} = \varepsilon^{r} + i\varepsilon^{i}$. Figure 4 shows the effect of the real part of permittivity of the core zone on the permittivity of cloak. We have set $\varepsilon^{r} = 2 \times n$ and $\varepsilon^{i} = 0.1$, $n$ is the magnification of the real part of permittivity, other parameters are the same as above. From the Fig. 4, we can find that with the increase of the parameter $\varepsilon^{r}$, both the real part and the imaginary part of the dielectric constant ${\upvarepsilon }_{{2}}^{{\text{a}}}$ decreases exponentially, and finally tend to be a stable value. However, the dielectric constant ${\upvarepsilon }_{{2}}^{{\text{b}}}$ shows different changing rules, and its real and imaginary parts both increase exponentially, but its real part does not tend to be stable. In addition, we have observed that with the increase of the cloak thickness, the real part of the permittivity ${\upvarepsilon }_{{2}}^{{\text{a}}}$ increases continuously, but its imaginary part and the permittivity ${\upvarepsilon }_{{2}}^{{\text{b}}}$ both decrease continuously. So, the requirement of cloak’s permittivity can be improved by adjusting its thickness, which makes it possible to design a practical cloak. In addition, we also can use material doping to obtain the proper dielectric constant^75,76^.

**Figure 5:**
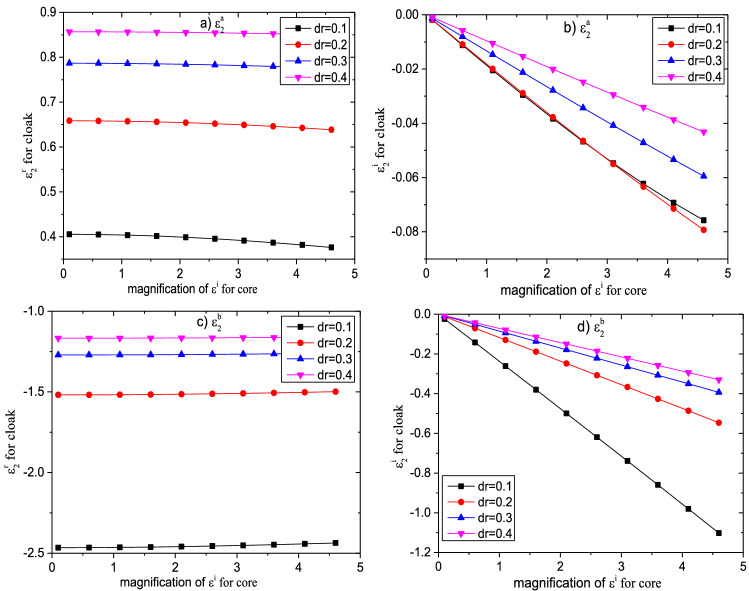
Influence of $\varepsilon^{i}$ on the permittivity of the cloak with different permittivity.

In Fig. 5 we make a similar discussion on the absorbent particle, with a permittivity $\varepsilon^{r} = 2,\varepsilon^{i} = n \times 0.1$, $n$ is the magnification of the imaginary part of core’s permittivity. Other parameters are the same as above. We find that with the increase of $\varepsilon^{i}$, the real part of ${\upvarepsilon }_{{2}}^{{\text{a}}}$ decreases linearly, while its imaginary part is increasing linearly. However, the dielectric constant of the cloak designed by the negative refractive material ${\upvarepsilon }_{{2}}^{{\text{b}}}$ still increases linearly. However, no matter which one material is used, the change degree of the real part of the dielectric constant of the cloak is much less than that of its imaginary part. Besides, we also found that when the ratio between the inner radius and outer radius of the core–shell particle remains the same, the change of particle geometry size does not affect the dielectric constant of the required shell (cloak) medium.

## Discussion

In summary, based on the theoretical derivation and numerical simulation, an equivalence relationship between positive-permittivity and negative-permittivity materials in electric invisibility cloak is proposed. We also present a formula to realize the conversion between the positive and negative permittivity of the corresponding materials. The numerical results show that both positive-permittivity and negative-permittivity materials all can be used to achieve an electric invisibility cloak, and the positive-permittivity cloak can reduce the electric field inside it, while the negative-permittivity cloak can enhance the electric field inside it. In addition, we find that the permittivity of cloak is influenced by the physical parameters of core and the thickness of cloak. In terms of the equivalence of real physical field, this idea is feasible, and it also can be applied to other types of physical fields. Moreover, especially and importantly, we have demonstrated the conversion relationship between the positive-permittivity and negative-permittivity dielectric materials, further research is needed to determine whether similar relationships exist in other physical fields.

## Methods

The distribution of electric potential inside and outside the particle can be represented as the following equations in the spherical coordinates^73^.

7 $$\phi_{1} = - AE_{0} r\cos \theta$$

8 $$\phi_{2} = - E_{0} (Br - Cr^{ - 2} )\cos \theta$$

9 $$\phi_{m} = - E_{0} (r - Dr^{ - 2} )\cos \theta$$

Supposed that $x = {{\left( {\varepsilon_{1} - \varepsilon_{2} } \right)} \mathord{\left/ {\vphantom {{\left( {\varepsilon_{1} - \varepsilon_{2} } \right)} {\left( {\varepsilon_{1} + 2\varepsilon_{2} } \right)}}} \right. \kern-\nulldelimiterspace} {\left( {\varepsilon_{1} + 2\varepsilon_{2} } \right)}},\xi = {{R_{1}^{3} } \mathord{\left/ {\vphantom {{R_{1}^{3} } {R_{2}^{3} }}} \right. \kern-\nulldelimiterspace} {R_{2}^{3} }}$, the parameters $A,B,C,D$ can be calculated through the following expressions^73^,

$$\begin{gathered} A = \frac{{9\varepsilon_{m} \varepsilon_{2} }}{{(\varepsilon_{2} + 2\varepsilon_{m} )(\varepsilon_{1} + 2\varepsilon_{2} ) + 2\xi (\varepsilon_{2} - \varepsilon_{m} )(\varepsilon_{1} - \varepsilon_{2} )}}\quad B = \frac{{3\varepsilon_{m} (\varepsilon_{1} + 2\varepsilon_{2} )}}{{(\varepsilon_{2} + 2\varepsilon_{m} )(\varepsilon_{1} + 2\varepsilon_{2} ) + 2\xi (\varepsilon_{2} - \varepsilon_{m} )(\varepsilon_{1} - \varepsilon_{2} )}} \hfill \\ C = \frac{{3\varepsilon_{m} (\varepsilon_{1} + \varepsilon_{2} )r_{2}^{3} }}{{(\varepsilon_{2} + 2\varepsilon_{m} )(\varepsilon_{1} + 2\varepsilon_{2} ) + 2\xi (\varepsilon_{2} - \varepsilon_{m} )(\varepsilon_{1} - \varepsilon_{2} )}}\quad D = \frac{{\xi (2\varepsilon_{2} + \varepsilon_{m} )x + (\varepsilon_{2} - \varepsilon_{m} )}}{{2\xi (\varepsilon_{2} - \varepsilon_{m} )x + (\varepsilon_{2} + 2\varepsilon_{m} )}}r_{2}^{3} \hfill \\ \end{gathered}$$

the equivalent dielectric constant $\varepsilon_{equ}$ of the core–shell particle also can be obtained,

10 $$\varepsilon_{equ} = \frac{1 + 2\xi x}{{1 - \xi x}}\varepsilon_{2}$$

In order to cancel the perturbation of the external field by the object, we need to make $\varepsilon_{equ} { = }\varepsilon_{m}$ and then a new equation derived,

11 $$\xi (2\varepsilon_{2} + \varepsilon_{m} )x + (\varepsilon_{2} - \varepsilon_{m} ) = 0$$

Then through some simplified operation we can obtain the Eq. (1).

In addition, if we add the Eqs. (4) and (5) together, then we can obtain,

12 $${\upvarepsilon }_{{2}}^{{\text{a}}} + {\upvarepsilon }_{{2}}^{{\text{b}}} = \frac{{ - 2\beta_{1} }}{{4\left( {1 - \xi } \right)}} = \frac{{ - \beta_{1} }}{{2\left( {1 - \xi } \right)}}$$

Then substitute the expression of $\beta_{1}$ in Eq. (12) we can obtain the Eq. (6).

The above derivation can also be applied to the cloak design in static magnetic fields, or the condition that the object size is much smaller than electromagnetic wavelength.
